# A Web-Based Therapist Training Tutorial on Prolonged Grief Disorder Therapy: Pre-Post Assessment Study

**DOI:** 10.2196/44246

**Published:** 2023-03-27

**Authors:** Kenneth Kobak, M Katherine Shear, Natalia A Skritskaya, Colleen Bloom, Gaelle Bottex

**Affiliations:** 1 Center for Telepsychology Madison, WI United States; 2 Columbia University School of Social Work New York, NY United States

**Keywords:** grief, prolonged grief disorder, evidence-based practice, mental health training, therapist training, new technology, web-based training, dissemination, e-learning

## Abstract

**Background:**

Prolonged grief disorder (PGD) is a newly recognized mental disorder characterized by pervasive intense grief that persists longer than cultural or social expectations and interferes with functioning. The COVID-19 epidemic has resulted in increased rates of PGD, and few clinicians feel confident in treating this condition. PGD therapy (PGDT) is a simple, short-term, and evidence-based treatment developed in tandem with the validation of the PGD diagnosis. To facilitate the dissemination of PGDT training, we developed a web-based therapist tutorial that includes didactic training on PGDT concepts and principles as well as web-based multimedia patient scenarios and examples of clinical implementation of PGDT.

**Objective:**

We aimed to evaluate user satisfaction with the tutorial and whether the tutorial increased trainees’ knowledge of PGDT principles and procedures. Moreover, we included a small number of pilot questions to evaluate the PGDT-related clinical skills.

**Methods:**

This study evaluated tutorial learning using a pre- and poststudy design. Participants were recruited from professional organization mailing lists, announcements to graduates of the Columbia School of Social Work, and through word of mouth. After signing consent, participants completed a brief demographic survey, a 55-item multiple-choice prestudy test on the concepts and principles of PGD and PGDT covered in the tutorial, and a 4-item pilot web-based prestudy test to gauge PGD clinical implementation skills. The link to the course content was then activated, and participants were given 8 weeks to complete the 11-module tutorial containing information, web-based exercises, simulated patient and video examples, and self-tests.

**Results:**

Overall, 406 clinicians signed consent, and 236 (58.1%) started the tutorial. Of these, 83.1% (196/236) completed all 11 modules. Trainee scores on our PDGT assessment improved substantially from pretraining to the postmodule assessment, with the total number of correct answers increasing from a mean of 29 (SD 5.5; 52.7% correct) to 36.7 (SD 5.2; 66.7% correct; *t*_195_=18.93; *P*<.001). In addition, the trainee’s implementation scores on 4 clinical vignettes increased from 2.6 (SD 0.7) correct out of 4 to 3.1 (SD 0.4) out of 4 (*t*_188_=7.02; *P*<.001). Effect sizes (Cohen *d*) were 1.44 (95% CI 1.23-1.65) for PDGT assessment and 1.06 (95% CI 0.84-1.29) for implementation. Trainees found the tutorial interesting, enjoyable, clearly presented, and useful for professional development. They endorsed a mean score of 3.7 (SD 0.47) on a 1 to 4 scale of agreement with recommending the course to others and feeling satisfied with the tutorial, and a mean of 3.3 (SD 0.57) with feeling able to apply the skills with clients.

**Conclusions:**

This pilot study provides support for the usefulness of this web-based training for teaching clinicians how to administer PGDT. The addition of patient scenarios for clinical implementation strategies holds promise for increasing the effectiveness of PGDT training and other evidence-based treatments.

**Trial Registration:**

ClinicalTrials.gov NCT05121792; https://www.clinicaltrials.gov/ct2/show/NCT05121792

## Introduction

### Background

Prolonged grief disorder (PGD) is a new diagnosis in the Diagnostic and Statistical Manual of Mental Disorders (DSM) fifth edition text revision [1], and the World Health Organization’s International Classification of Diseases 11th edition (ICD-11) in 2019 [2]. PGD is characterized by persistent pervasive intense grief that interferes with functioning for a period that exceeds expectations of the person’s social, cultural, or religious groups and is at least 6 months after the loss in the ICD-11 and at least 12 months in the DSM fifth edition. The disorder is characterized by persistent intense yearning, longing, or preoccupation with the person who died, accompanied by at least 3 of 8 associated symptoms, also persistent and pervasive to a clinically significant degree and occurring daily for at least the past month: the loss of a sense of identity, marked sense of disbelief about the death, avoidance of reminders that the person has died, intense emotional pain such as anger or sadness related to death, difficulty reengaging with others or with one’s own life, a feeling of emotional numbness, feeling that life is meaninglessness because of death, or intense loneliness as a result of death. Studies have found that PGD is associated with major impairment in social, occupational, and leisure activities [3,4]; increased risk for suicide [5] (rates higher than depression [6]); and negative health consequences, for example, cancer and cardiovascular disease [3].

The introduction of a new mental disorder begs the question of available treatments. In the case of PGD, PGD therapy (PGDT; formerly called Complicated Grief Therapy) was developed [7] and tested in 3 randomized controlled trials with a total of 641 participants [8-10] before the inclusion of PGD as an official diagnosis. This treatment research initiative paralleled and contributed to the research that validated the criteria for new diagnosis [11,12]. Each of the 3 studies, sponsored by the National Institute of Mental Health, compared PGDT to a proven efficacious treatment for depression, either interpersonal psychotherapy in 2 of the studies or antidepressant medication in the third. PGDT produced an average response rate of 71%, compared with 33% for depression treatment. Importantly, depression is most often confused with PGD [8,9,13].

### PGDT Overview

**Figure 1:**
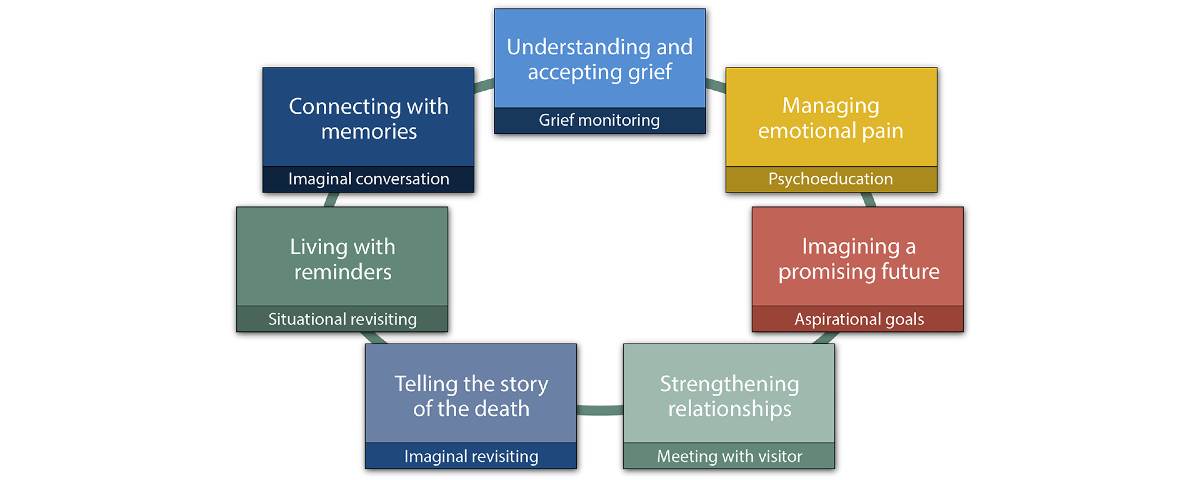
Themes and processes in prolonged grief therapy.

PGDT is a short-term (16 sessions) integrated psychotherapy targeting adaptation to loss. PGDT is based on research-informed principles and evidence-based methods from cognitive behavioral therapy, interpersonal psychotherapy, motivational interviewing, positive psychology, and psychodynamic psychotherapy. Attachment theory is used to understand bereavement and grief and to define the treatment goal of facilitating adaptation to loss. Following the dual-process model of coping with bereavement [14], adaptation is conceptualized as entailing the acceptance of the reality of the loss and restoration of the capacity for well-being. The foundational premises of PGDT are that grief is a stress response and a form of love that emerges naturally and finds a place in our life. Although everyone grieves and adapts in their own way, there are commonalities. Adapting to loss progresses naturally if it does not become derailed. Derailers are naturally present during early grief and can get in the way of adapting if they persist over time and gain too much prominence in mental functioning [15]. PGD therapists use active listening and personalized interventions, as they work through a planned sequence of sessions and a series of well-specified psychological exercises. These exercises provide experiential learning opportunities for each of the 7 themes that operationalize the process of adapting to loss, understanding and accepting grief, managing grief-related emotions, seeing a future with promise, strengthening relationships, narrating a coherent story of death, living with reminders, and feeling connected with memories (Figure 1). The first 2 themes help patients understand and manage grief; the next 2 focus on restoring the capacity for well-being using the self-determination theory goals of autonomy, competence, and relatedness [16]. The last 3 themes help patients to accept the reality of the loss and establish a sense of connection with the person who died. For more detailed information, refer to the study by Shear [16].

The critical shortage of clinicians trained in evidence-based treatment has been amplified by the pandemic. The rate of psychiatric disorders has increased [17], with more patients seeking help [18]. This has already put a strain on clinicians who are unlikely to be knowledgeable about PGD or PGDT. This means that a large number of therapists will need to be trained in a short amount of time. One way to facilitate access to training is through the use of digital technologies.

Internet-based training, both synchronous and asynchronous, can be available to any clinician with internet access. Asynchronous training is unconstrained by enrollment limitations, trainer availability, and time limitations, as busy clinicians can work at their own pace [19]. Using the principles of instructional design, such as high interactivity and multimodal learning, enhances the quality of training and increases knowledge retention [20]. Web-based technologies have been used to successfully train clinicians in several evidence-based treatments such as interpersonal psychotherapy for depression [21], cognitive therapy for adolescent depression [22], anxiety disorders [23], and drug abuse [24]. Web-based training has also been used to help train non–mental health clinicians to deal more effectively with mental health issues, such as emotional trauma [25] and adolescent mental health [26]. While no web-based therapist training for treating grief has been reported, several web-based self-help interventions for grief have been published [27-30].

In a review of the use of technology to train clinicians in evidence-based treatments, Singh and Reyes-Portillo [31] found that technology-based training can be just as effective as traditional training and has the potential to facilitate the adoption of evidence-based practice. Fairburn and Wilson [32] suggested that internet-enhanced technologies might provide the only scalable solution to the challenge of disseminating clinician training on evidence-based treatments as well as supporting the actual use of skills in clinical settings after completing training. Internet-based training can also provide opportunities for ongoing updates, specific steps to prevent drift, retesting, and monitoring. In addition, we have had positive experiences combining synchronous and asynchronous training methods.

A major issue in training clinicians on evidence-based treatments is not only how to facilitate the uptake of conceptual knowledge but also how to teach and assess effective clinical implementation of the treatment. Several studies have found that web-based training is more effective when followed up by clinical consultation [33-35]. However, live supervision is costly, time-consuming, and not easily scalable. To help make this aspect of training scalable, German et al [36] used a *train the trainer* approach, where a cohort of clinicians within a setting were trained by experts, using in-person workshops followed by live supervision. Once trained, these clinicians used their expertise to train new clinicians within the group [36] using a combination of web-based training followed by live supervision. They found that the combination of web and live training was as effective in producing clinical competency as training performed entirely by clinicians. Murphy et al [37] used web-based video playback technology (mPath) to help trainees reflect upon their interpersonal counseling skills in specific therapy sessions. In our previous studies, we used live remote observation via videoconference for training on applied skills using standardized patients [38] or trainers playing the parts of the patient while providing live feedback [21,23,39,40]. These studies found that live remote training improved not only didactic knowledge but also applied clinical skills and led to successful treatment outcomes [22,23]. However, this approach is still costly and does not solve the scalability problem.

In the tutorial, we included a small pilot component to address clinical implementation strategies using web-based multimedia patient scenarios with animated vignettes or previously produced actors with scripted videos. This approach allows for repetitive practice and immediate feedback in a safe and structured environment. To pilot this effort, users observed an actor-therapist interacting with an actor-patient (or an animated therapist interacting with an animated patient). This scenario portrayed a challenging clinical situation that clinicians might encounter when performing PGDT. Users indicated one of several possibilities for how they would respond to a situation. Through practice and feedback, clinicians can learn ways to engage in similar conversations with actual patients. The use of similar actor-patient scenarios to train clinicians has been reported for suicide risk assessment [41] and the treatment of major depression [42], alcohol [43], and substance use disorders [44].

### Aim of This Study

The purpose of this study was to report on trainees’ experiences and their learning outcomes after completing our recently released web-based therapist training tutorial on PGD and PGDT. Specifically, we measured (1) trainee satisfaction with the tutorial, (2) improvement in trainee knowledge of the principles and procedures used in PGDT, and (3) improvement in trainee choice of clinical implementation strategies in delivering PGDT.

## Methods

### Study Participants

Participants were therapists with a mental health–related degree or graduate students in a mental health program, recruited between October 2021 and May 2022. Study participants were recruited from electronic mailing lists of licensed psychologists in New York State, announcements to persons on e-newsletter lists from the Columbia Center for Prolonged Grief, announcements to individuals who had previously taken a course at the Center for Telepsychology, announcements to graduate students at the Columbia School of Social Work, and by word of mouth. Interested individuals reviewed the web-based information sheet consent form and indicated that they freely agreed to participate in the study. Those agreeing to participate were provided a username and password and linked to a brief demographic survey, a 55-item multiple-choice pretest on the principles and concepts of PGD and PGDT, and a 4-item web-based pretest to assess PGD clinical implementation strategies. Once the demographic survey and pretests were completed, the link to course content was activated.

### Description of PGDT Tutorial

The web-based PGDT tutorial contains didactic information, web-based exercises, simulated patient scenarios, animated graphics, ongoing web-based self-tests, and video examples of patient role-plays in a multimodal, multimedia, and web-based learning approach that research has found to enhance learning efficacy [20] (Multimedia Appendix 1).

The tutorial contains 11 modules covering the following topics: the nature of grief, an overview of PGD and PGDT, pretreatment assessment, a module for each of the 7 PGDT themes, and a final module that provides a summary of the treatment progress and addresses treatment termination. As PGDT is a *measurement-based* approach (ie, an intervention that includes regular structured assessment with simple validated instruments) [45], the tutorial reviews the assessment tools used in PGDT and how to integrate them into treatment. Each module is approximately 20 to 40 minutes long.

Trainees work through the tutorial at their own pace. However, they are encouraged to space out the time they work on it rather than take it in a few long sessions, as spaced learning increases knowledge retention [46]. A posttest is given immediately after completing each module, as testing performed closer to when the material was learned improved retention [47]. Consistent with continuing education guidelines, successful completion requires an overall score of 80% on the posttests. Users can retake the module until a passing score is obtained. The participants in this study were given 8 weeks to complete the training.

### Study Assessment Instruments

#### Evaluation of User Satisfaction

We used three measures to evaluate user satisfaction with this tutorial: (1) rates of course completion, (2) scores on a satisfaction questionnaire, and (3) ratings on whether course objectives were achieved.

##### Course Completion

Course completion was defined as the completion of all 11 modules, including the quizzes. The number of dropouts per week and study module was also examined.

##### User Satisfaction Questionnaire

User satisfaction was evaluated using an 8-item User Satisfaction Questionnaire. This scale is similar to that used in prior web-based clinician training studies [38,48-53].

##### User Rating of Learning Objectives

User satisfaction was also evaluated based on the percentage of trainees who felt that the learning objectives of the tutorial were met. The learning objectives for each module are stated at the beginning of each module. There were 46 learning objectives across the 11 modules. After completing each module, the trainees were asked to indicate whether they agreed that the specific learning objectives were met on a scale of 1 to 4 (1=strongly disagree, 2=disagree, 3=agree, and 4=strongly agree).

#### Evaluation of Trainee Knowledge of Principles and Procedures Used in PGDT

We developed a set of 55 questions, including 5 questions related to each of the 11 training modules. The participants answered a 55-item questionnaire at baseline before being given access to the tutorial. They were then asked to answer 5 of these questions at the end of each tutorial module (Multimedia Appendix 2). After posting their answer to each question, the participants were given feedback with the best answer and rationale. Finally, to progress to the next module, trainees were required to repeat any of the 5 items until they correctly answered at least 4 of the 5 questions. We used the first answer for all questions as the end-point measure in the analyses described here. However, repeated testing with feedback is part of the educational process; thus, we believe this is a conservative estimate of the participants’ actual learning.

#### Evaluation of Trainee Clinical Implementation Strategies in Delivering PGDT

We developed a series of animated vignettes and used previously produced scripted role-play videos using actors that demonstrated a series of challenging scenarios. After viewing a video segment, the trainee was presented with several possible therapist responses and asked to select one that they thought would be best. After providing their answers, they received feedback on their choices. To evaluate whether this experience influenced the trainee’s implementation strategies in delivering PGDT, we used four intervention choice points as a pre- and posttest: (1) a discussion during review of grief monitoring, (2) a patient questioning the value of monitoring grief, (3) a scenario in which there was a derailer present in a discussion of aspirational goals, and (4) setting up an avoidance hierarchy for situational revisiting. These are examples of common kinds of challenges a therapist might encounter in PGDT (Multimedia Appendix 3).

### Statistical Methods

This study used a pre- and poststudy design. Paired *t* tests (2-tailed) were used to measure the pre- to posttest changes in PGDT knowledge and clinical implementation strategies. The effect sizes (Cohen *d*) were calculated to examine the magnitude of the changes. The McNemar chi-square test was used to test the differences in percentages. Descriptive statistics were calculated for user satisfaction and learning objectives data.

### Ethics Approval and Informed Consent

The study was reviewed and approved by the Columbia University Institutional Review Board on October 13, 2021 (registration number: AAAT7389). The approval process also obliged us to register this study at ClinicalTrials.gov. All participants provided written informed consent before being given access to the tutorial.

## Results

### Demographic and Professional Characteristics of Tutorial Users

The demographic characteristics of the participants who completed the tutorial are presented in Table 1. The sample was primarily female (178/196, 90.8%) and White (159/196, 81.1%), with a mean age of 48.9 (SD 13.7; range 22-79) years. The sample predominantly included social workers and psychologists with a smattering of other mental health professions. Therapists had a mean of 2.7 (SD 11.0; range 0-44) years’ experience doing therapy. Most (115/196, 58.7%) reported having had prior grief training, and 85.7% (168/196) reported having personally experienced grief.

**Table 1:** Demographic and professional characteristics of the participants (n=196).

Characteristics			Values
Age (years), mean (SD)			49.9 (13.7)
**Sex, n (%)**			
	Female	178 (90.8)	
	Male	15 (7.6)	
	Intersex or other	3 (1.5)	
**Self-identified race, n (%)**			
	White	159 (81.1)	
	Black or African American	11 (5.6)	
	Asian	8 (4.1)	
	Mixed race	4 (2)	
	Other	14 (7.2)	
**Hispanic identity, n (%)**			
	Non-Hispanic	173 (88.3)	
	Hispanic	23 (11.7)	
**Highest educational degree, n (%)**			
	Bachelor’s	10 (5.1)	
	Associate	2 (1)	
	Master’s	131 (66.8)	
	Doctoral	45 (23)	
	Other	3 (1.5)	
**Profession, n (%)**			
	Social worker	89 (45.4)	
	Psychologist	71 (36.2)	
	Physician	5 (2.6)	
	Mental health counselor	5 (2.6)	
	Nurse	2 (1)	
	Clergy	2 (1)	
	Marriage and family counselor	1 (0.5)	
	Physician’s assistant	1 (0.5)	
	Graduate students	16 (8.2)	
	Other	4 (2)	
Years of experience conducting psychotherapy, mean (SD)			12.7 (10)
**Prior grief training, n (%)**			
	Yes	136 (69.4)	
	No	60 (30.6)	
**Personally experienced a loss and grief, n (%)**			
	Yes	168 (85.7)	
	No	25 (12.8)	
	Prefer not to answer	3 (1.5)	

### User Satisfaction

#### Tutorial Completion

A total of 6538 recruitment emails were sent. Of these, 6.2% (406/6538) expressed interest in the study and signed consent forms within a few days. At that point, we closed recruitment, as the study enrollment goals were met. Of those who signed the consent form, 58.1% (236/406) completed the questionnaires and began the tutorial. Of the 236 who started the tutorial, 196 (83.1%) completed it. The numbers of dropouts per module are listed in Table 2. Module completion rates fell gradually but minimally from 236 to 196, that is, from module 1A and 1B and then from 2 to 11.

**Table 2:** Pre- to posttest improvement in participants’ knowledge of prolonged grief disorder therapy concepts by module (score range per module is 0-5).

Tutorial module	Values, n	Prestudy test, mean (SD)	Poststudy test, mean (SD)	*t* test (*df*)	*P* value
All 10 modules	196	29 (5.5)	36.7 (5.2)	18.93 (195)	<.001
1A. The nature of grief	236	2.5 (1.2)	4.1 (0.9)	17.1 (225)	<.001
1B. Overview of prolonged grief disorder	227	1.7 (1.0)	2.7 (1.3)	10.1 (225)	<.001
2. Pretreatment assessment	213	2.4 (1.1)	4.3 (0.9)	23.7 (212)	<.001
3. Understanding and accepting grief	210	2.6 (1.1)	3.5 (1)	10.8 (208)	<.001
4. Managing emotional pain	208	2.7 (0.9)	3.5 (0.9)	9.8 (206)	<.001
5. Imagining a promising future	203	2.6 (1.1)	3.4 (1.1)	7.5 (201)	<.001
6. Strengthening relationships	200	3.1 (1.2)	4.2 (0.8)	11.6 (198)	<.001
7. Telling the story of the death	200	2.4 (1.1)	3.8 (1)	14 (199)	<.001
8. Living with reminders	199	2.3 (1)	4.0 (1)	19.2 (197)	<.001
9. Connecting with memories	198	1.5 (1)	3.4 (1.2)	19.1 (197)	<.001
10. Putting the treatment together and managing its ending	196	2.6 (0.9)	2.8 (0.7)	2.3 (195)	.031

#### User Satisfaction Questionnaire

Trainees (n=192) scores on the 8 user satisfaction questionnaires were uniformly high. They scored between 3=agree and 4=fully agree on 6 of the 8 questions, including material presented in an interesting manner, concepts presented clearly and easy to understand, would recommend this course to others, enjoyed taking the tutorial, and felt able to apply these skills to clients. They said that they learned a lot and found information useful for their practice. Finally, they endorsed the feeling that the learning objectives were achieved (Multimedia Appendix 4).

#### Learning Objectives

The average learning objective was reported as being met (ie, “agree or strongly agree”) by 96.9% (182/188) of clinicians. A list of all 46 learning objectives, their mean ratings, and percentage of trainees rating the objective as being met can be found in Multimedia Appendix 5.

### Evaluation of Trainee Knowledge of Principles and Procedures Used in PGDT

**Figure 2:**
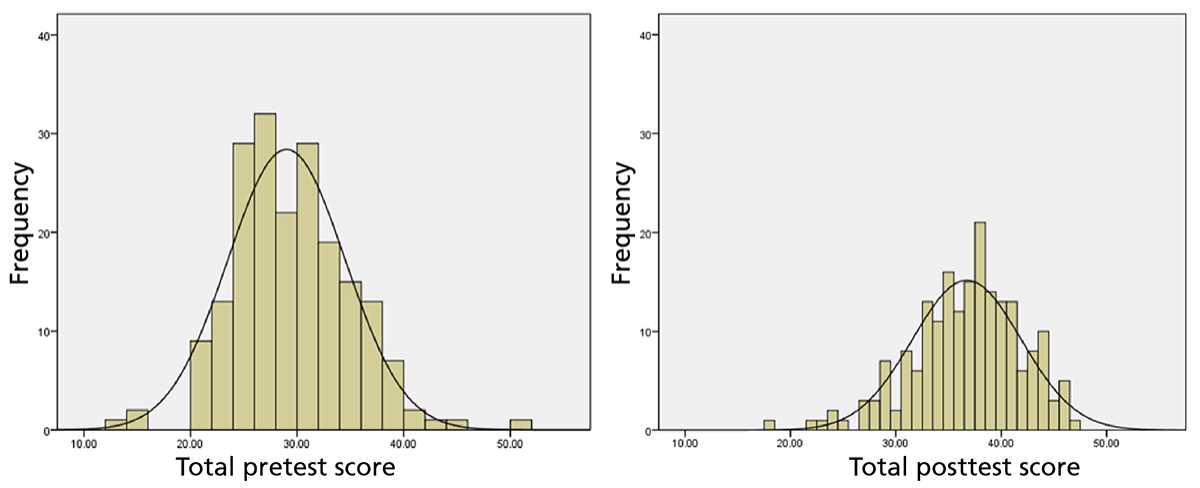
Histogram of pre- and posttest scores on prolonged grief disorder treatment concepts and procedures.

As shown in Table 2, trainee scores on PGDT concepts and procedures ratings showed statistically significant improvement from pretraining to the postmodule assessment, with the total number of correct answers increasing from 29 (SD 5.5; 53% correct) to 36.7 (SD 5.2; 67% correct; t_195_=18.93; *P*<.001; Figure 2; effect size: Cohen *d*=1.44; 95% CI 1.23-1.65). The largest increases in scores were found in modules 2 (pretreatment assessment) and 8 (living with reminders), and the smallest increase was found in module 10 (putting the treatment together and managing treatment ending). While those with prior grief training scored slightly higher on the pretest than those without prior grief training, 29.9 (53%) versus 27.6 (50%; t_194_=2.93, *P*=.004), the mean change was 7.7 for both groups. Not surprisingly, there was a difference between the mean change for graduate students (n=16; 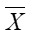 1=5.8; SD 5.9) and licensed clinicians (n=179; 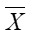 1=7.9, SD 5.7; t_194_=1.41, *P*=.19).

### Evaluation of Trainee Clinical Implementation Strategies in Delivering PGDT

The mean change in trainees’ scores on the pilot clinical implementation assessment increased substantially, from 2.6 (SD 0.7; 65%) correct out of 4 to 3.1 (SD 0.4; 78%) out of 4 (t_188_=7.03; *P*<.001). The standardized effect size was *d*=1.05 (95% CI 0.84-1.29). The changes in each response are presented in Table 3. There was no major difference in this measure between those with and without prior grief training (t_186_=0.98; *P*=.32) or between clinicians and graduate students (t_193_=1.40; *P*=.16).

**Table 3:** Pre- to posttest improvement in prolonged grief disorder therapy clinical decision-making skills (n=188).

Skill	Participants prestudy test (%)	Participants poststudy test (%)	Chi-square test (*df*)	*P* value
1. Reviewing grief monitoring	70.4	95.2	40.1 (1)	<.001
2. Questioning the value of monitoring grief	89.9	98.9	15.2 (1)	<.001
3. Dealing with derailers in aspirational goals	12.73	14.3	0.5 (1)	.64
4. Setting up a fear hierarchy for situational revisiting	92.6	97.98	6.3 (1)	.01

## Discussion

### Principal Findings

The principal findings of this study support the growing body of literature supporting the helpfulness of asynchronous web-based technologies for training clinicians in evidence-based treatments [31,54,55]. Scores on trainee ratings on our PGDT assessment measures increased substantially, as, to a lesser extent, did our trainee clinical implementation strategy assessment. In addition, the high response rate to our recruitment notices, as well as the unusually large percentage who completed the tutorial, suggests a high level of interest in this tutorial. Higher user satisfaction was further supported by the User Satisfaction Questionnaire scores and the percentage of trainees reporting that the learning objectives were met. The high completion rate we obtained was notable and may be related to the appeal of this program, including appealing interactivity, novelty, animated and video clinical examples, and the novelty of a new DSM diagnosis that already has a well-validated treatment approach. User satisfaction is especially important in asynchronous web-based training, where there is a high risk of discontinuing training.

Given the recent introduction of PGD in ICD-11 and DSM fifth edition text revision, as well as a marked increase in persons with prolonged grief owing to the pandemic, rapid dissemination of training on effective PGD treatment is needed. Over 6 million people died worldwide during the pandemic. A total of 1.1 million people died of COVID-19 in the United States [56], adding substantially to the 2.8 million deaths per year. A recent US demographic study estimated that each death leaves about 9 bereaved relatives [57], which suggests that the bereaved outnumber the deceased by nearly 10-fold [58]. Overall, the rates of PGD among all bereaved individuals have been estimated to be approximately 10% [59], with higher rates for more difficult deaths. Circumstances of pandemic deaths have been especially challenging and thus qualify as particularly difficult deaths that are likely to be associated with elevated rates of PGD.

Notably, 85.7% (168/196) of the trainees reported having experienced a major loss. Many therapists become interested in grief therapy after experiencing their own grief. Life experience is a good way to understand the experience of a patient, so this may be a benefit. Alternatively, a personal loss may sensitize a therapist in a way that might make therapy more challenging. However, we found no difference in responses to this tutorial among those who did or did not report an important grief experience.

Therapists with prior grief training scored substantially higher on our pretest than those without this training, but the difference was only a few points. The scores of both groups showed a reliable increase on our assessment questionnaire after taking the tutorial. This suggests that even experienced therapists and those with prior personal grief experience or prior grief training can benefit from this training on PGD and its treatment. However, a mean score of 67% indicates that more learning is required to achieve optimal training results.

We included 4 pilot questions related to the use of PGDT clinical implementation skills, which mostly showed improvements in these assessments, suggesting that this is a promising extension of web-based training that might be further developed. Live role-plays with immediate feedback and clinical supervision may still be the best way to support the development of clinician skills [33-35]. However, given that PGD is a new disorder, there are not yet sufficient numbers of PGDT trainers to meet the needs of a large number of clinicians needing this training, and the observation of the trainee’s role-plays (either live or remote) is not yet scalable. Web-based technology can offer a useful tool to augment didactic training. Web-based patient scenarios provide an alternative in which trainees may gain comfort in implementing and experimenting with new skills. This may be a more comfortable way to receive feedback on their performance, especially in the early stages of training. Future work should continue to build methods for the effective asynchronous practice of clinical skills.

### Study Limitations

This study has several limitations. First, we recruited individuals through internet announcements to a wide range of professionals, but those who signed up were mostly more experienced therapists, and the majority had already had grief training. It is important to know how therapists who are less comfortable or knowledgeable about grief would respond to this tutorial. Second, perhaps the most important limitation of this study is that there are no patient outcomes and no measures of therapist adherence when providing therapy. Thus, it is unclear whether training improves treatment efficacy. Third, although we showed large pre-post effect sizes in our measured outcomes, our conclusions are limited by the lack of information about the reliability and validity of the test items. In addition, posttests were conducted immediately after the material was presented. Whether the knowledge gained was retained is unknown. Fourth, the therapists in our sample were primarily White (159/196, 81.1%). Most concerning, only 6.1% (21/196) self-identified as Black compared with the population prevalence of 13% in the United States and the elevated rates of both yearly death rates and PGD among people of color. This low rate may be due to failure to reach Black professionals; failure of Black therapists to be interested in the tutorial; or perhaps most concerning, a low level of Black professionals trained as therapists. A secondary analysis of our most recent intervention efficacy study showed no difference in response rates between individuals who self-identified as White or Black. Clearly, more work is needed to ensure that efficacious evidence-based treatment is available to people of different cultures [60].

### Conclusions

This preliminary study provides support for the effectiveness of web-based training in teaching clinicians to recognize PGD and administer PGDT. The inclusion of a web-based multimedia tutorial for didactic training and simulated patient scenarios to develop PGDT-related clinical skills holds promise for increasing the effectiveness of web-based training. The model and components used in this tutorial model may also be helpful in the dissemination of training for other evidence-based treatments. Web-based training may help facilitate training in evidence-based treatments by overcoming barriers owing to limited trainer capacity, time, and scheduling constraints [61,62]. Further studies are warranted to determine the reliability and validity of the tutorial outcome measures and explore the optimal use of web-based training in the context of other approaches to PGDT training. Such studies would provide data that would enable a cost-benefit analysis of the best ways to integrate each approach into the training of grief therapists.

## Appendix

Multimedia Appendix 1 Screenshots of video examples, web-based exercises, simulated patient scenarios, web-based self-tests, and animated graphics.

Multimedia Appendix 2 Examples of items on the 55-Item multiple-choice pre- and posttest of prolonged grief disorder therapy knowledge.

Multimedia Appendix 3 Web-based example of reviewing grief monitoring.

Multimedia Appendix 4 Mean satisfaction ratings of web-based tutorials in the User Satisfaction Questionnaire.

Multimedia Appendix 5 Trainee ratings on whether learning objectives were met by module.

## Abbreviations

DSM: Diagnostic and Statistical Manual of Mental Disorders
ICD-11: International Classification of Diseases 11th edition
PGD: prolonged grief disorder
PGDT: prolonged grief disorder therapy

## Data Availability

Deidentified data files are available on Dropbox over internet [63].
