# Supplementary material for: A Web-Based Therapist Training Tutorial on Prolonged Grief Disorder Therapy: Pre-Post Assessment Study
Source: JMIR Med Educ. 2023 Mar 27;9:e44246. doi: 10.2196/44246 (PMC10131787; doi:10.2196/44246)
Supplement: Multimedia Appendix 1 [file mededu_v9i1e44246_app1.doc]

Multimedia Appendix 1

Screen Shots of Video Examples, Interactive Exercises, Simulated Patient Scenarios, Interactive Self-Tests, and Animated Graphics

This is a Multimedia Appendix to a full manuscript published in the J Med Internet Res. For full copyright and citation information see <http://dx.doi.org/10.2196/jmir.44246>.

**Screen Shot: Video Illustration of Initial Interview**


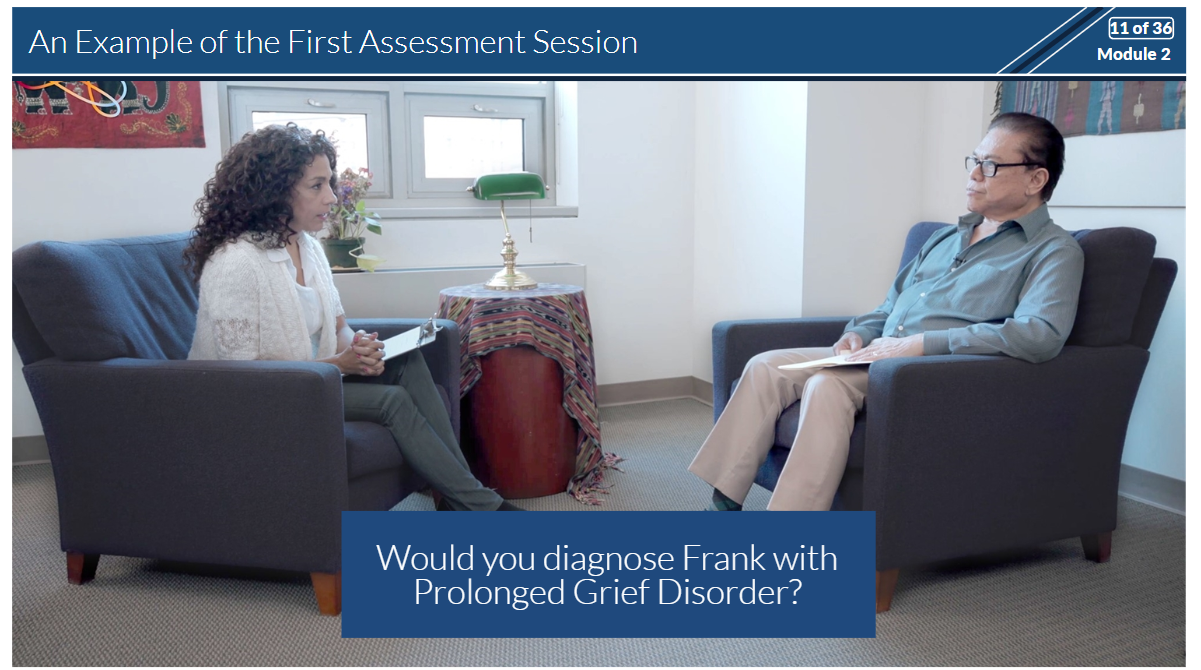


Frank is a 65-year-old retired police officer. His son Jack was 33 and recently married when a tragic accident took his life 18 months ago. Frank feels like he died that night too. He’s sure all the joy in his life is gone forever and he almost wants it that way - even though he has a daughter and 2 grandchildren. People told him time would be healing, but it’s not true. If anything, he’s getting worse. He feels like an automaton, disconnected from everything, even his family. He takes care of what has to be done getting no sense of satisfaction. He made this appointment because his wife said he had to.

Listen to the following initial intake session. As you listen, try to identify any possible derailers. Decide if you think you would diagnose Frank with Prolonged Grief Disorder and whether or not you would recommend prolonged grief disorder therapy. Click on the video to begin.

**Screen Shot: Interactive Exercise**


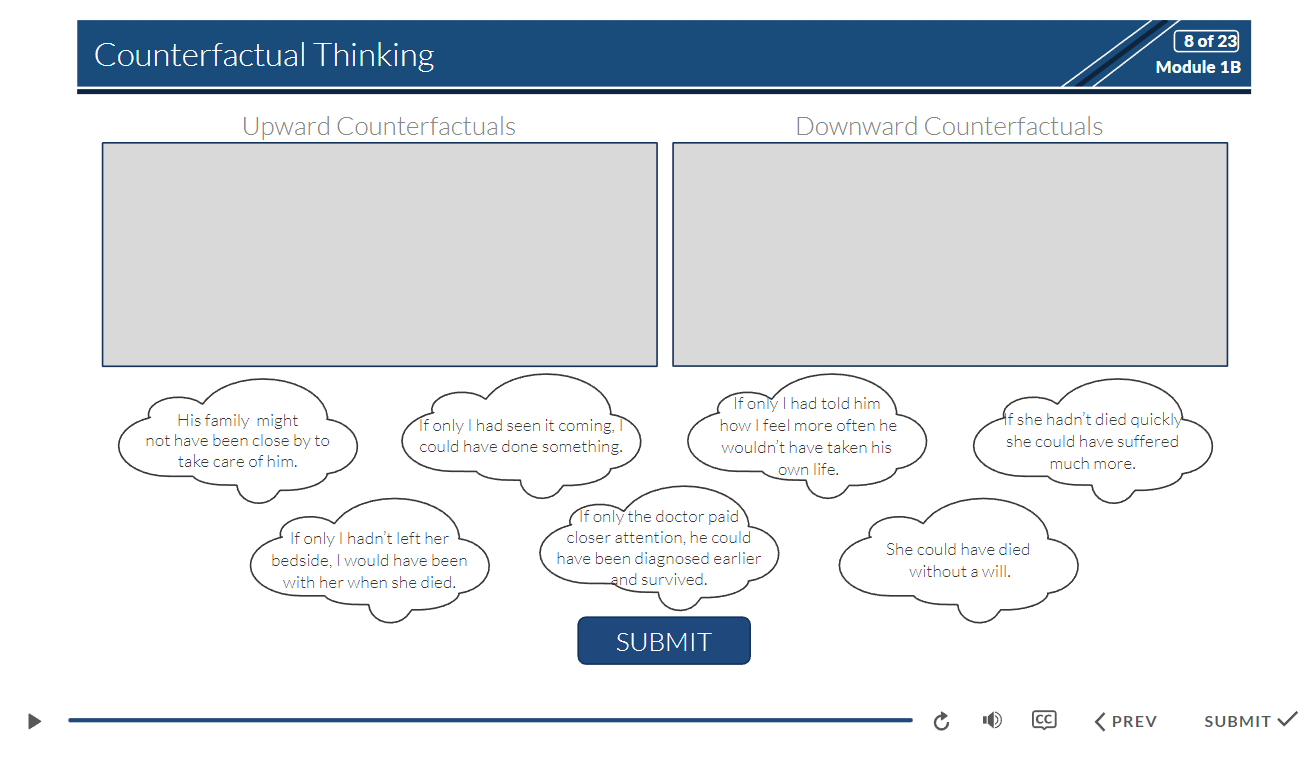


Drag each statement to the correct box, then click Submit to see how you did.

**Screen Shot: Simulated Patient Scenario**


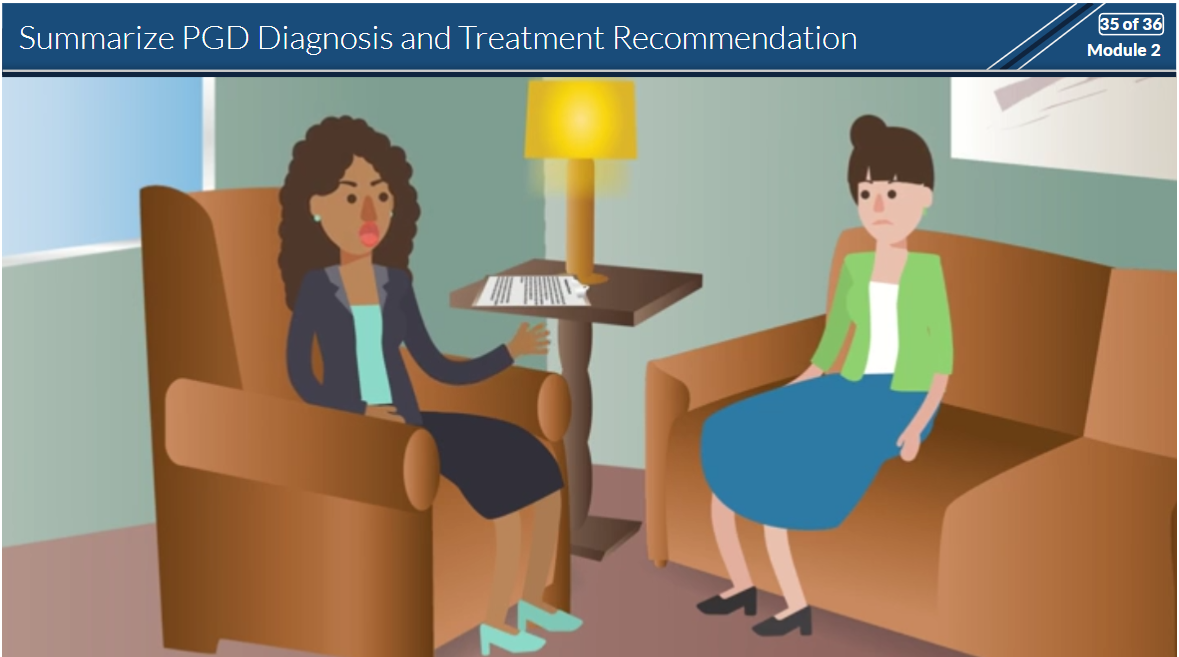


Click to see an example of a therapist summarizing the initial session.

**Screen Shot: Interactive Self-Test**


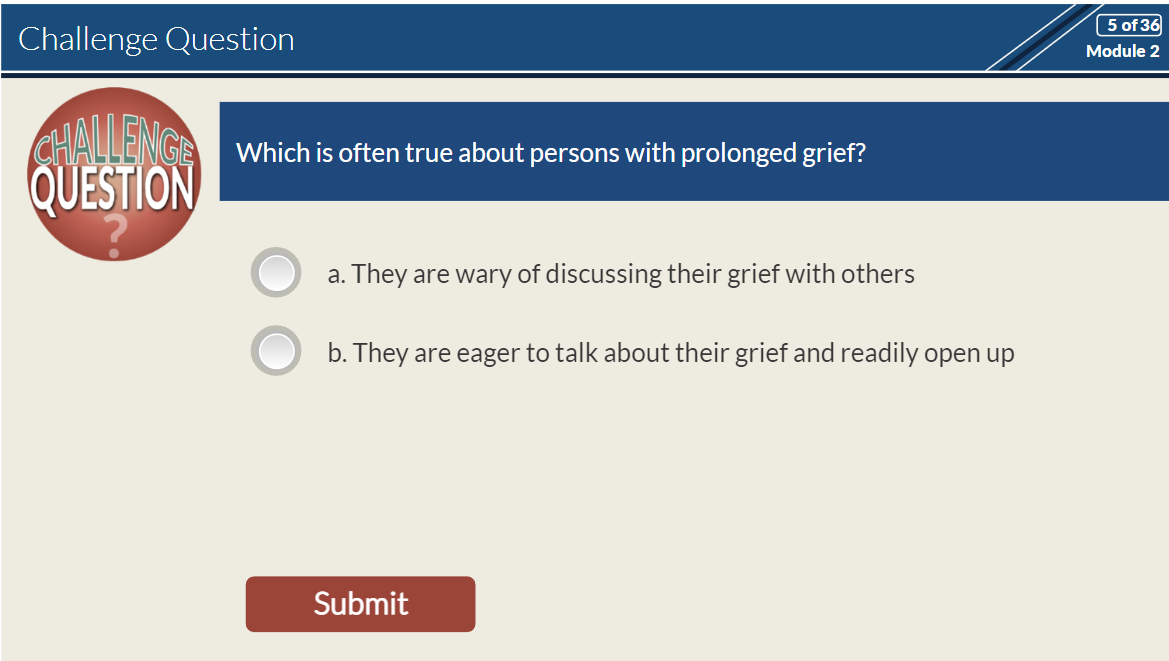


**Screen Shot: Animated Graphics**


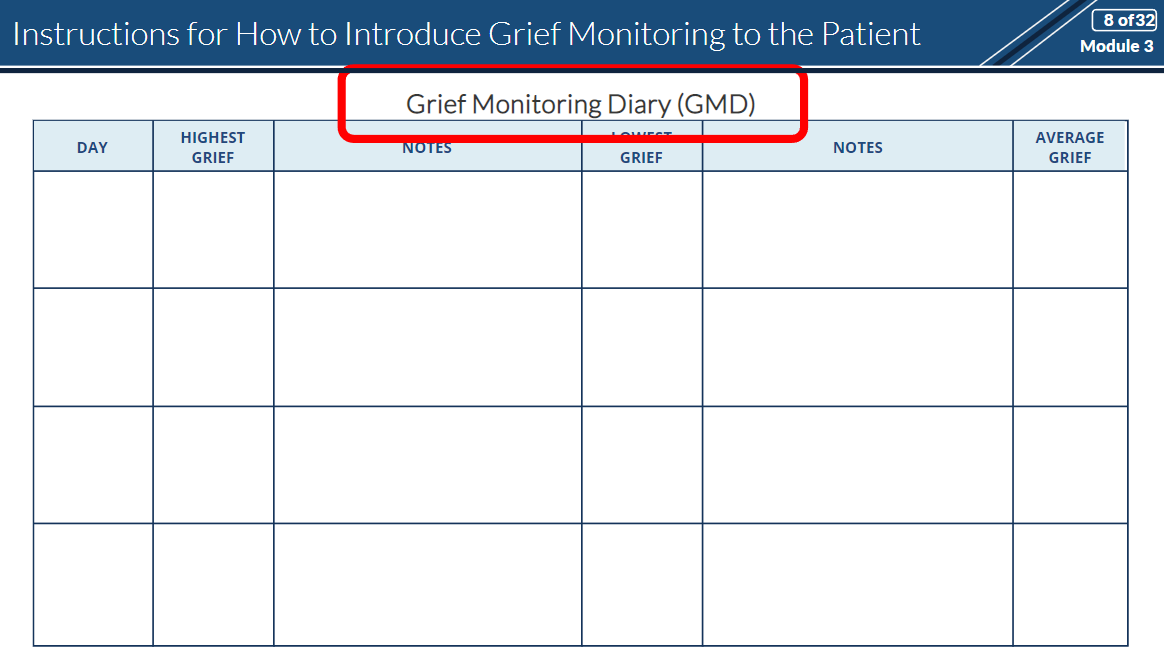


**Begin by giving the patient a Grief Monitoring Form and asking them to take a look at it. Tell them you want them to start paying attention to their grief and how it varies in intensity on a daily basis. They don’t need to take a lot of time for this-usually about 5 minutes at the end of each day.**


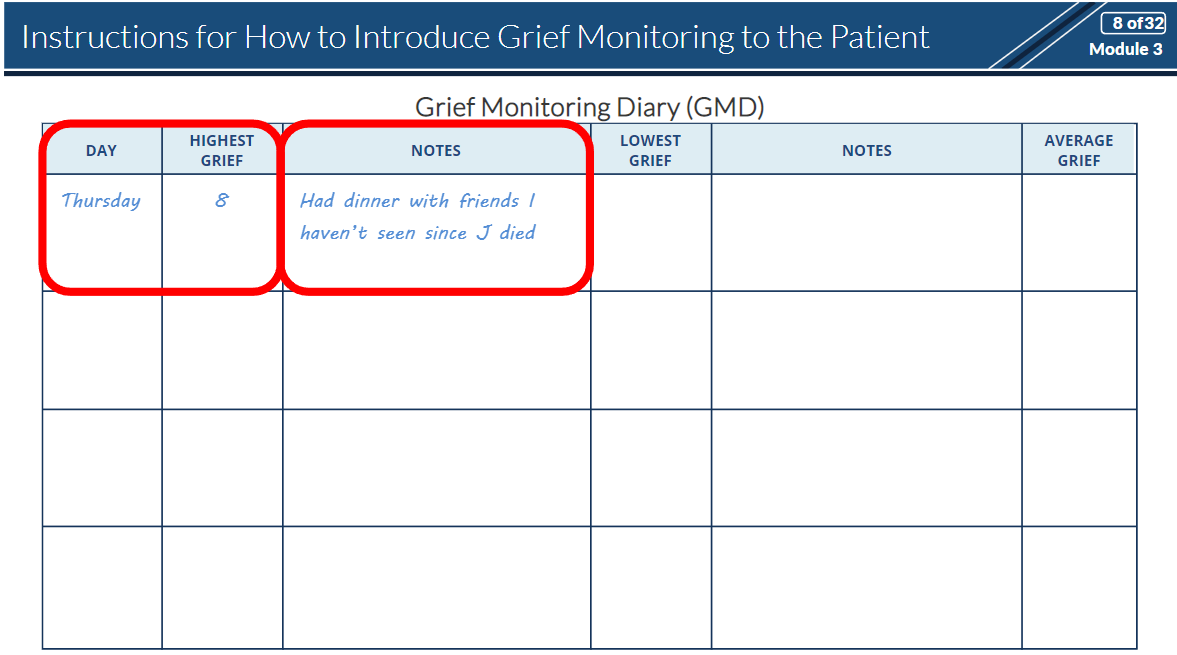


**First, they identify a time when their grief was highest for that day, rate the intensity on a scale from 1-10 and make a note about what was happening at that time. If there are a number of times when it was at its highest, they select one of those times.**


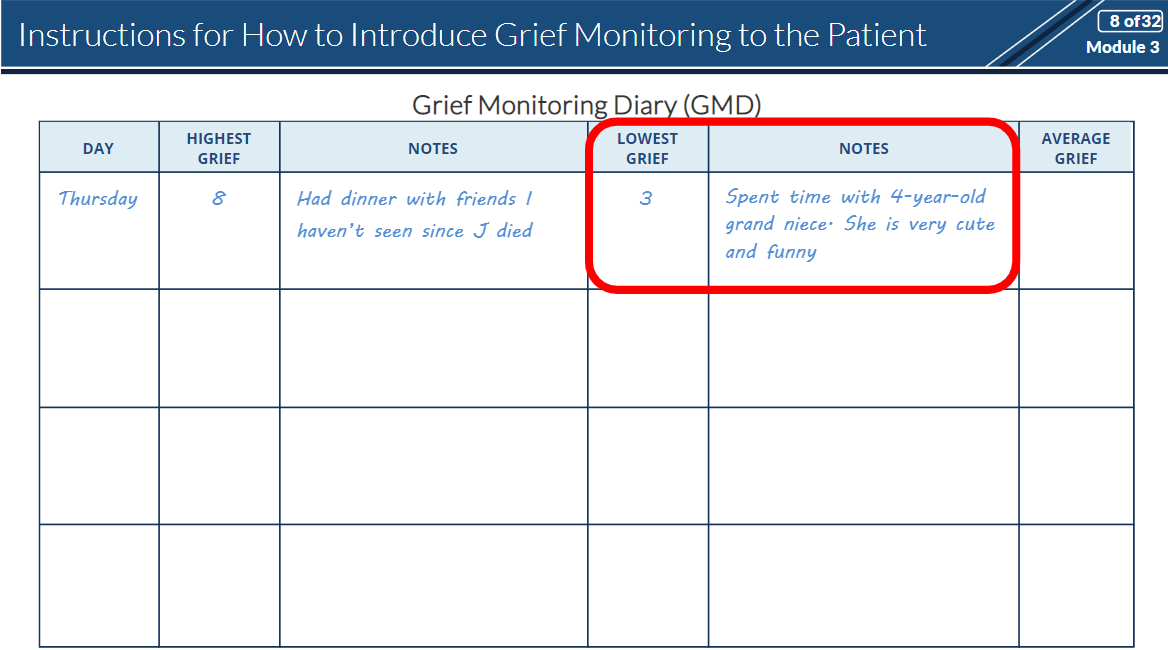


**They repeat this process for the lowest level of grief that day.**


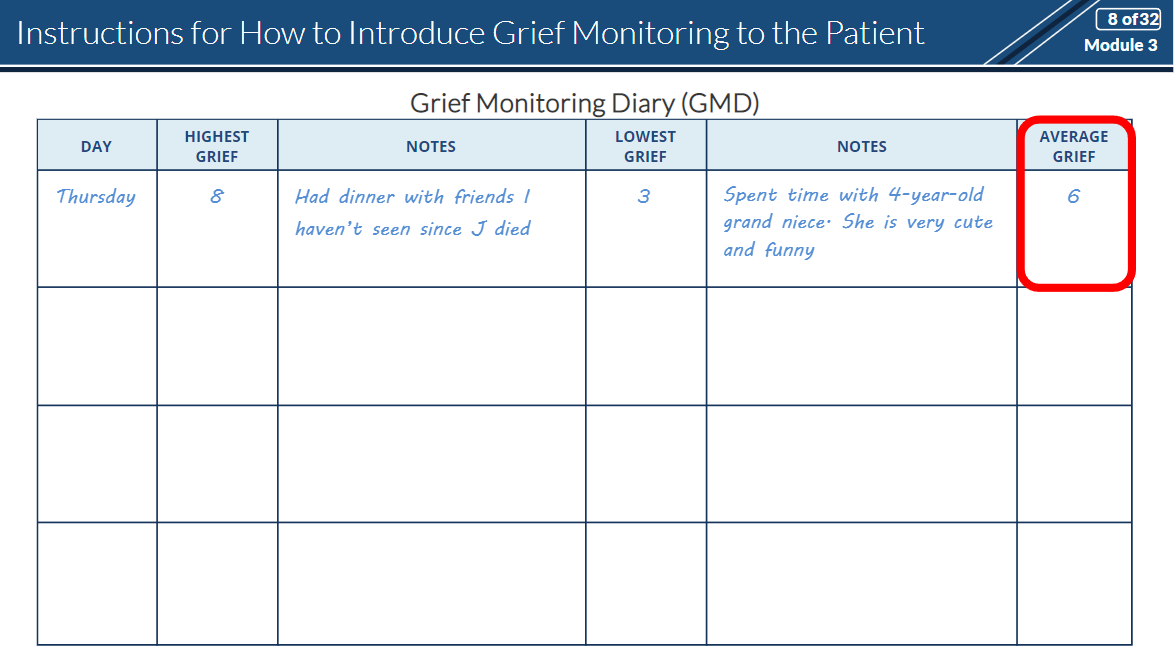


**Then think about the day, overall. Was their overall grief level high, moderate or low? Then rate high as somewhere between 7-10, moderate as 4-6 and low as 1-3 on a scale of 1-10.**


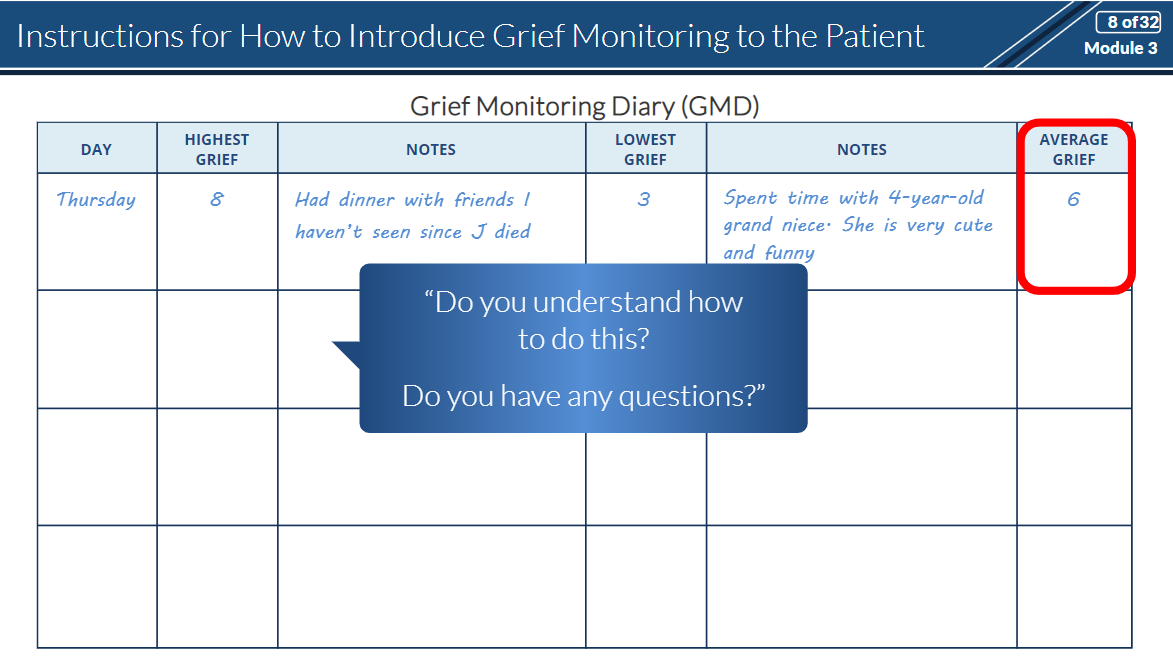


**Ask if the patient understands how to do this and if they have any questions.**
