# Supplementary material for: A Web-Based Therapist Training Tutorial on Prolonged Grief Disorder Therapy: Pre-Post Assessment Study
Source: JMIR Med Educ. 2023 Mar 27;9:e44246. doi: 10.2196/44246 (PMC10131787; doi:10.2196/44246)
Supplement: Multimedia Appendix 2 [file mededu_v9i1e44246_app2.doc]

Multimedia Appendix 2

Examples of items on the 55-Item Multiple Choice Pre- and Post-test of PGDT Knowledge

This is a Multimedia Appendix to a full manuscript published in the J Med Internet Res. For full copyright and citation information see <http://dx.doi.org/10.2196/jmir.44246>.

**Module 1A: What it means to lose a loved one**

1. Which of the following are part of attachment theory of close relationships:
2. Caregiving system, i.e., taking care of others
3. Exploratory system, i.e., our interest in learning and growing
4. An internal working model of the relationship in our implicit and explicit memory
5. A and C
6. All of the above

**Module 1B: Overview of Prolonged Grief and Prolonged Grief Therapy**

1. Which might be barriers to adapting to loss of a loved one?
   1. Counterfactual (if only) thoughts related to the death
   2. Experiencing too much grief
   3. Experiencing too little grief
   4. All of the above

**Module 2: Pretreatment Assessment**

1. Which of the following are true about major depression and prolonged grief
2. Effective treatments for depression do not relieve symptoms of prolonged grief
3. Adding medication to prolonged grief therapy in patients with co-occurring major depression improves outcomes
4. Both of the above

**Module 3 Quiz Greif Monitoring**

1. A patient asks if they can expect their grief level to go down if treatment is working. Your best response would be:
2. It’s important not to have expectations about your grief level. It’s fine to let it be whatever it is
3. While there are no guarantees, reassure the patient that there is empirical data that shows that CGT treatment is effective
4. The purpose of grief monitoring is to help you understand your grief, not change it
5. A and C
6. All of the above

**Module 4: Psychoeducation**

1. Which of the following are true of psychoeducation?
   1. It helps clients understand the goals of therapy
   2. It helps clients believe the treatment makes sense
   3. It helps the therapeutic alliance
   4. A and b
   5. All of the above

## Module 5: Aspirational Goals & Rewarding Activities

## The rationale for work on aspirational goals include:

## It helps the patient connect to things they find truly interesting or things they really care about

## It helps generate a sense of enthusiasm for the future and motivation to accept loss and grief

## Patient with complicated grief may be confused about what they care about, or feel they don’t care about anything anymore

## A and B

## All of the above

**Module 6: Session with a significant other**

1. Which of the following are goals of the session with a significant other?
2. To help the visitor understand complicated grief and complicated grief therapy
3. To learn the visitor’s perspective on the patient
4. To enlist the significant other’s help in encouraging the patient to complete treatment and treatment exercises
5. B and C
6. All of the above

**Module 7: Imaginal Revisiting**

1. The following are goals of the imaginal revisiting procedure:
   1. Gain confidence in managing emotional pain associated with telling this story
   2. Strengthen the capacity for self-observation and reflection
   3. Develop a coherent narrative and come to terms with the reality of the death
   4. Learn that Rewarding activities can help in managing emotional pain
   5. A, b and C
   6. All of the above

**Module 8: Situational Revisiting**

1. The rationale for situational revisiting includes
2. Avoiding situations that are reminders of the loss often deprives the patient of meaningful memories.
3. Confronting situations with painful reminders provides an opportunity to enjoy these situations in a new way
4. Repeatedly confronting triggers provides an opportunity to find additional ways to manage these emotions
5. a and c
6. All of the above

# Module 9: Memories Forms and Imaginal Conversation

# Which of the following are true about memories in persons with complicated grief?

# They typically don’t have access to positive memories

# They have positive memories, but they serve to increase their pain

# Their positive memories, tend to occur in a dissociative state

# B and C

**Module 10: Putting the treatment together**

1. Which of the following do you need to remain mindful of as a therapist throughout treatment?
   1. Personalizing interventions to address derailers and/or adapting to loss
   2. Monitoring your role as a temporary secure attachment
   3. Observing your own fear of loss being triggered
   4. a and c
   5. All of the above
