# Supplementary material for: A Web-Based Therapist Training Tutorial on Prolonged Grief Disorder Therapy: Pre-Post Assessment Study
Source: JMIR Med Educ. 2023 Mar 27;9:e44246. doi: 10.2196/44246 (PMC10131787; doi:10.2196/44246)
Supplement: Multimedia Appendix 3 [file mededu_v9i1e44246_app3.doc]

Multimedia Appendix 3

Interactive Example of Reviewing Grief Monitoring

This is a Multimedia Appendix to a full manuscript published in the J Med Internet Res. For full copyright and citation information see <http://dx.doi.org/10.2196/jmir.44246>.

To access, click on the link below:

[**http://telepsychology.net/grieftherapy/story.html**](http://telepsychology.net/grieftherapy/story.html)
