# Supplementary material for: A Web-Based Therapist Training Tutorial on Prolonged Grief Disorder Therapy: Pre-Post Assessment Study
Source: JMIR Med Educ. 2023 Mar 27;9:e44246. doi: 10.2196/44246 (PMC10131787; doi:10.2196/44246)
Supplement: Multimedia Appendix 4 [file mededu_v9i1e44246_app4.doc]

Multimedia Appendix 4

Mean satisfaction ratings of on-line tutorial on the User Satisfaction Questionnaire

This is a Multimedia Appendix to a full manuscript published in the J Med Internet Res. For full copyright and citation information see <http://dx.doi.org/10.2196/jmir.44246>

| **Item** | **Item Range** | **Mean (SD)** |
| --- | --- | --- |
| 1. The material was presented in an interesting manner | 1-4 | 3.6 (0.55) |
| 2. The concepts were clearly presented and easy to understand | 1-4 | 3.6 (0.50) |
| 3. I would recommend this course to others | 1-4 | 3.7 (0.46) |
| 4. I enjoyed taking this tutorial | 1-4 | 3.6 (0.57) |
| 5. I feel able to apply these skills with clients | 1-4 | 3.3 (0.57) |
| 6. Overall, how satisfied were you with this tutorial? | 1-4 | 3.7 (0.47) |
| 7. How much did you learn as a result of this tutorial? | 1-7 | 6.3 (0.79) |
| 8. How useful was the content of the tutorial for your practice or professional development? | 1-5 | 4.6 (0.61) |

Note: Items 1-4, Scale = 1=strongly disagree, 2=disagree, 3=agree, 4=strongly agree

Item 6: 1=very dissatisfied, 2=dissatisfied, 3=satisfied, 4=very satisfied.

Item 7: 1= very little to 7=a great deal

Item 8: 1=not useful to 5=extremely useful

Note: N=192
