# Supplementary material for: A Web-Based Therapist Training Tutorial on Prolonged Grief Disorder Therapy: Pre-Post Assessment Study
Source: JMIR Med Educ. 2023 Mar 27;9:e44246. doi: 10.2196/44246 (PMC10131787; doi:10.2196/44246)
Supplement: Multimedia Appendix 5 [file mededu_v9i1e44246_app5.doc]

Multimedia Appendix 5

Trainee Ratings on Whether Learning Objectives Were Met by Module

This is a Multimedia Appendix to a full manuscript published in the J Med Internet Res. For full copyright and citation information see <http://dx.doi.org/10.2196/jmir.44246>.

| **Module** | Mean (SD) | Agree or Strongly Agree (%) |
| --- | --- | --- |
| **1A. The nature of grief**  Describe grief as a complex multifaceted response to loss  Explain how close relationships affect us and how this helps explain grief  Describe grief-related emotions, cognitions and behavior  Analyze coping with loss-related stress and adapting to loss-related changes | 3.4 (0.8)  3.5 (0.6)  3.5 (0.6)  3.4 (0.5) | 92%  97%  97%  97% |
| **1B. Overview of Prolonged Grief Disorder and Prolonged Grief Therapy**  Describe prolonged grief disorder and how it relates to DSM5 and ICD11 criteria for Prolonged Grief Disorder  Describe Healing milestones and derailers and their role in complicated grief treatment  Explain risk factors that increase the likelihood of derailers  Explain the approach used in prolonged grief disorder therapy (PGDT) | 3.6 (0.6)  3.5 (0.6)  3.5 (0.6)  3.5 (0.6) | 98%  96%  97%  98% |
| **2. Pre-treatment assessment**  Describe the rationale and procedure for a 2-session pre-treatment assessment of a bereaved patient  Explain key elements of establishing an alliance with a bereaved patient,  Explain how to make a treatment-relevant DSM5 diagnosis of PGD  Describe how to introduce complicated grief therapy to a patient diagnosed with PGD | 3.7 (0.5)  3.6 (0.5)  3.6 (0.6)  3.5 (0.5) | 100%  99%  97%  98% |
| **3. Grief monitoring**  Explain the procedure, goals and rationale for grief monitoring in PGDT  Analyze how and when to use grief monitoring in PGDT  Introduce grief monitoring to a patient  Review a grief monitoring form after a patient completes it  Discuss how grief monitoring can be used to address derailers | 3.4 (0.6)  3.5 (0.6)  3.6 (0.6)  3.6 (0.6)  3.4 (0.6) | 97%  97%  98%  99%  95% |
| **4. Psychoeducation**  Explain the procedure, goals and rationale for psychoeducation  Analyze how and when to use psychoeducation in PGDT  Explain Prolonged Greif Disorder and Prolonged Grief Disorder Therapy to a patient  Analyze how psychoeducation can be used to help patients address derailers | 3.5 (0.5)  3.4 (0.5)  3.4 (0.5)  3.3 (0.6) | 99%  98%  99%  95% |
| **5. Aspirational goals and rewarding activities**  Explain the, goals and rationale for aspirational goals and rewarding activities in PGDT  Analyze how to work on aspirational goals and rewarding activities in PGDT  Troubleshoot difficulty in working on aspirational goals and rewarding activities | 3.5 (0.6)  3.4 (0.6)  3.3 (0.6) | 98%  97%  96% |
| **6. Session with a significant other**  Explain the goals, rationale and procedure, for a session with a significant other in PGDT  Analyze ways to achieve the goals of a session with a significant other in PGDT  Describe how to conduct a session with a significant other in session 3  Explain how to follow up with strengthening relationships throughout the treatment  Troubleshoot hesitancy or difficulty in holding a session with a significant other | 3.5 (0.5)  3.4 (0.6)  3.5 (0.5)  3.3 (0.7)  3.4 (0.6) | 99%  98%  99%  96%  97% |
| **7. Imaginal revisiting**  Explain the procedure, goals and rationale for Imaginal revisiting in PGDT  Describe how to introduce Imaginal revisiting in session 4  Explain how to conduct Imaginal revisiting in sessions 5-9  Troubleshoot difficulties in working on Imaginal revisiting | 3.4 (0.6)  3.4 (0.6)  3.4 (0.6)  3.3 (0.6) | 97%  98%  97%  94% |
| **8. Situational revisiting**  Explain the procedure, goals, and rationale for situational revisiting  Describe how to introduce situational revisiting  Explain how to follow up with situational revisiting throughout the remaining treatment  Troubleshoot hesitancy or difficulty in working on situational revisiting | 3.5 (0.5)  3.4 (0.5)  3.4 (0.5)  3.3 (0.5) | 99%  99%  99%  96% |
| **9. Memory forms and imaginal conversation**  Explain the goals, rationale and procedure for using memories forms  Explain the goals, rationale and procedure for imaginal conversation  Describe when to introduce memories forms and imaginal conversation  Troubleshoot hesitancy or difficulty in working on memories forms or imaginal conversation | 3.5 (0.5)  3.5 (0.5)  3.5 (0.6)  3.2 (0.6) | 99%  99%  97%  94% |
| **10. Putting the treatment together and managing its ending**  Explain how to link the CGT model, themes, procedures, and goals in the 16-session model  Analyze how to keep track of yourself, your patient, and your goals during a session  Describe how and when to use a group of interventions, beyond the seven specific CGT procedures  Explain how to sequence within and across the 16 sessions, and describe what the patient does between sessions  Describe the goals and procedures used in CGT termination | 3.3 (0.6)  3.3 (0.6)  3.1 (0.6)  3.3 (0.6)  3.4 (0.5) | 97%  95%  84%  95%  98% |

Note: Scale = 1=strongly disagree, 2=disagree, 3=agree, 4=strongly agree
